# Nanomaterials Synthesis through Microfluidic Methods: An Updated Overview

**DOI:** 10.3390/nano11040864

**Published:** 2021-03-28

**Authors:** Adelina-Gabriela Niculescu, Cristina Chircov, Alexandra Cătălina Bîrcă, Alexandru Mihai Grumezescu

**Affiliations:** 1 Faculty of Engineering in Foreign Languages, University Politehnica of Bucharest, 060042 Bucharest, Romania; niculescu.adelina19@gmail.com; 2 Faculty of Applied Chemistry and Materials Science, University Politehnica of Bucharest, 060042 Bucharest, Romania; cristina.chircov@yahoo.com (C.C.); ada_birca@yahoo.com (A.C.B.); 3 Research Institute of the University of Bucharest—ICUB, University of Bucharest, 050657 Bucharest, Romania

**Keywords:** microfluidic devices, microfluidic technology, nanoparticle synthesis, microreactors, nanomaterials

## Abstract

Microfluidic devices emerged due to an interdisciplinary “collision” between chemistry, physics, biology, fluid dynamics, microelectronics, and material science. Such devices can act as reaction vessels for many chemical and biological processes, reducing the occupied space, equipment costs, and reaction times while enhancing the quality of the synthesized products. Due to this series of advantages compared to classical synthesis methods, microfluidic technology managed to gather considerable scientific interest towards nanomaterials production. Thus, a new era of possibilities regarding the design and development of numerous applications within the pharmaceutical and medical fields has emerged. In this context, the present review provides a thorough comparison between conventional methods and microfluidic approaches for nanomaterials synthesis, presenting the most recent research advancements within the field.

## 1. Introduction

Nanotechnology gained significant importance when scientists realized that the size of the material is a major factor that influences the properties of a substance. Since then, several conventional methods have been employed for nanomaterials production, including condensation, chemical precipitation, and hydrothermal synthesis as the most common approaches [1,2,3]. Choosing an appropriate synthesis method with accurate control over the reaction conditions is essential for delivering high-quality products destined for specific applications. In this respect, a promising new technology emerged: microfluidics [4].

As one of the most prominent figures in the field of microfluidics, George Whitesides, stated, microfluidics is “the science and technology of systems that process or manipulate small (10^−9^ to 10^−18^ L) amounts of fluids, using channels with dimensions of tens to hundreds of micrometers” [5,6,7].

Microfluidic devices’ dimensions and unique geometries allow for smaller reagent volume use, precise control of fluid mixing, efficient mass transport, improved heat transfer, ease of automation, and reduced reaction time [7,8,9,10,11,12,13]. The advantages of using microfluidic methods over traditionally known approaches led to the design, fabrication, and usage of portable, low-cost, and disposable devices [6,9,14].

All the characteristics mentioned above make microfluidics highly advantageous for diverse applications, ranging from chemical, biological, and material industries to the pharmacy, clinical diagnosis, translational medicine, and drug discovery [11,14,15]. Being able to overcome some of the most challenging downsides of scale-up reactors, microfluidic technology is increasingly used in preparing nanoparticles and in carrying out various chemical syntheses [7,16]. It should be noted that microfluidic devices are also found in the literature under the term “microreactors” when used as synthesis vessels [7,10,14].

This work presents nanomaterials synthesis from the perspective of conventional and microfluidic methods, the advantages and challenges of each category, and the possible products they may yield. Thus, it provides a thorough comparison between traditional methods and microfluidic approaches, describing the most recent advancements and applications within the field.

## 2. Conventional Methods of Nanomaterials Synthesis

Nanomaterials are structures that have at least one dimension between 1 and 100 nm [17,18]. Such materials have revolutionized many domains, out of which the most intensively researched are related to modern medicine, especially regarding biosensors, diagnostics, targeted drug delivery, and therapeutics [19,20,21,22,23,24,25,26,27,28,29,30,31,32,33,34,35,36,37]. Having such a broad spectrum of applications, nanomaterials should be synthesized as efficiently as possible in order to gain extensive market reach.

**Figure 1:**
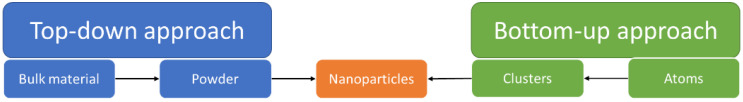
Nanoparticles synthesis approaches. Adapted from an open-access source [40].

Nanostructure formation can be achieved using two main approaches: top-down and bottom-up [16,38,39] (Figure 1). As the name implies, the top-down approach is based on the size-reduction of larger structures by means of mechanical force. Such methods are preferred for industrial scale-up, but they require expensive equipment and intensive energy without providing control over particle growth. By contrast, the bottom-up approach involves the growth and self-assembly of nanostructures from atomic or molecular precursors. Generally, this method results in the production of amorphous particles with increased solubility and bioavailability, which, however, tend to agglomerate. Nonetheless, such methods are simple, rapid, and energy- and cost-efficient, ideal for laboratory-scale production and synthesis of smaller particle sizes with narrow particle size distribution [7,16].

A variety of techniques are available for the synthesis of nanostructures (Table 1). Despite their diversity, these conventional approaches lack tight control over experimental variables, generating nanoparticles with wide size distribution and large inter-batch variability [41]. The poor selectivity of batch reactors results in their mediocre performance in terms of synthesizing products with controllable structures and properties [42].

Physical and chemical processes may provide uniform-sized nanoparticles yet at the expense of negatively impacting the environment. In other words, such techniques release toxic/hazardous materials into the environment [71,72], acting as pollutant sources and high-energy consumers [73]. Moreover, the need for large spaces, expensive equipment, and high-power consumption translates into high costs [7,71,72,74]. Other industrial scale-up issues include alternation of synthesis conditions and insufficient control of the mixing process during the preparation of nanoparticles [75], complex stepwise operations, waste of resources, poor reproducibility, safety concerns [42], highly specialized and difficult to manufacture equipment, and long synthesis times [76]. In addition to the disadvantages associated with the synthesis process, the obtained products may also suffer from uncontrolled particle growth (narrow size distribution shifted to large particle dimensions [77]), potential contamination [7], non-proper surface structures [72], and poor size distribution (high polydispersity index values) [42], which further affect the functionalities of the materials. Such limitations contribute to the hampering of synthetic chemistry from evolving towards green synthesis, big data, chemo/bioinformatics, and precision biomedicine [42]. Moreover, the limitations of conventional synthesis techniques result in a slow translation from research to practical applications, especially in the medical field [78,79,80]. Therefore, it is an urgent matter to develop an easy to manipulate technique for the efficient synthesis of high-quality nanomaterials [4].

## 3. Nanomaterial Synthesis via Microfluidic Approaches

Microfluidic technology provides the means to overcome some of the most pressing drawbacks of conventional synthesis methods due to the small capillary dimension and the resulting large surface-to-volume ratio. Through these features, rapid and uniform mass transfer and superior control over the produced nanomaterial characteristics are enabled in microfluidic syntheses [75]. In comparison to bulk methods, highly stable, uniform, monodispersed particles with higher encapsulation efficiency can be obtained by efficiently controlling the geometries of the microfluidic platform and the flow rates of the involved fluids [81].

As previously mentioned, microfluidic devices’ working principle is based on the movement of fluids within micro-scaled channels and chambers of special geometry, integrating sample preparation, reaction, separation, and detection [38,82].

**Figure 2:**
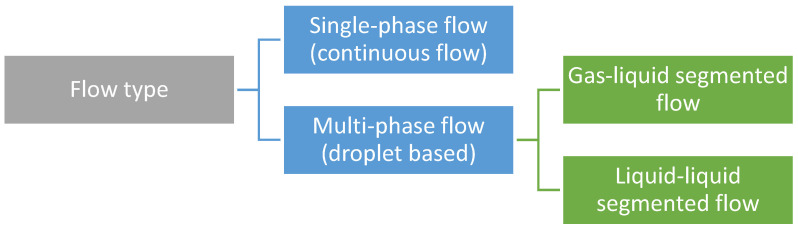
Microfluidic techniques classification. Created based on information from the literature [11,16,83].

Concerning synthesis strategies, there are two main types of microreactors depending on flow pattern manipulation, namely single-phase (continuous-flow microfluidics) and multi-phase flow (droplet-based microfluidics) (Figure 2) [16,83]. Each of these categories is further described in more detail.

### 3.1. Single-Phase Flow (Continuous-Flow) Systems

When it comes to nanoparticle production within microfluidic devices, single-phase systems are the most commonly used. This pattern flow is the variant of choice in many studies due to its simplicity, homogeneity, and versatility in controlling process parameters, such as flow, reagent amount, reaction time, and temperature [7,11].

Generally, single-phase synthesis is performed under laminar flow (with a Reynolds number lower than 10). Due to the absence of turbulence, the main mixing mechanism is molecular interdiffusion [7,11,16]. Therefore, continuous flow microfluidics is an excellent solution for nanoprecipitation processes, improving controllability, reproducibility, and homogeneity of product characteristics [7,84]. Therefore, the homogeneous environment present in single-phase flow systems is ideal for the synthesis of small nanoparticles with a narrow particle size distribution, which is especially needed in pharmaceutics formulations [7,83].

Nonetheless, molecular interdiffusion is a slow process, limiting reaction speed [85]. Moreover, single-phase flow reactors have a parabolic velocity profile that causes a nonuniform residence time distribution [86]. This velocity profile becomes problematic in the case of nanomaterials for which crystallization kinetics is sensitive to the residence time distribution in the early stages of growth, causing the nanoparticles flowing near the walls to have larger dimensions than those flowing near the center [16]. However, these drawbacks can be overcome by creating turbulence through bending/folding and stretching the microchannels, thus enhancing mixing [7,11].

### 3.2. Multi-Phase Flow (Droplet-Based) Systems

Unlike single-phase microfluidics, multi-phase flow (also known as segmented flow) systems involve two or more immiscible fluids [11]. Such heterogeneous systems facilitate passive mixing by enhancing mass transfer, narrowing the deviation of residence time and minimizing the deposition of reagents/products on channel walls [11,16,84].

As the name implies, droplet-based microfluidics concerns the formation and manipulation of discrete droplets inside microchannels [87]. Droplet production is regulated through device geometry, channel dimensions, and flow rates of each fluid, allowing precise monitoring and control over material fabrication processes [88,89].

There are two subcategories of multi-phase flow: gas–liquid (bubbles) and liquid–liquid segmented flows [16]. Gas–liquid segmented flow microfluidics is of interest due to the simple separation of gas from liquid, which can be useful for nanoparticle synthesis [11,84]. Another feature specific to gas–liquid flow systems is carrying reactions in segmented liquid slugs, where segmenting gas is introduced to create recirculation and to enhance mixing efficacy [11,84]. Bubbles can be created either by using active methods (e.g., short high-voltage pulses [85], acoustic micro streaming [90,91], and liquid metal actuators [92]) or in a passive manner (by simply bubbling a gas [93,94]). Through these methods, a microfluidic channel’s roughness is exploited towards rapid mixing and homogenization of the fluids [91]. Depending on the gas and liquid superficial velocities, annular flow patterns can also be employed. Such patterns appear when there is a continuous gas core flow in the channel center and a liquid film on the channel’s inner surface [16]. In liquid–liquid segmented flow systems, segmentation is achieved through surface tension differences between the immiscible fluid streams [16]. The flow patterns are often presented as water-in-oil or oil-in-water dispersions, requiring the addition of surfactants to minimize coalescence of the dispersed droplets [11].

Due to rapid production and analysis, droplets can be employed when developing reproducible and scalable particles with specific sizes, shapes, and morphologies, which are difficult to achieve otherwise [88,89]. In addition, droplet microreactors show enhanced mass and heat transport, accurate manipulation, reliable automation, and greater production capacity [7,10]. Hence, there is no surprise that multi-phase flow systems have become indispensable tools in various science applications [89]. These devices find use in producing emulsions, microdroplets, microparticles, and nanoparticles with distinct morphologies [7,88]. Moreover, droplets can act as single reaction vessels for cell growth [95].

However, several downsides to multi-phase flow systems must be considered when designing these applications. One of the disadvantages is the poor stability of droplets. This can be overcome through the addition of surfactants, but this solution is not suitable for all situations. Another issue comes from the fact that droplets are never completely isolated, as almost always, an extent of material exchange between droplets takes place [89]. However, whether these problems affect the desired outcome or not depends on what the device is used for.

**Figure 3:**
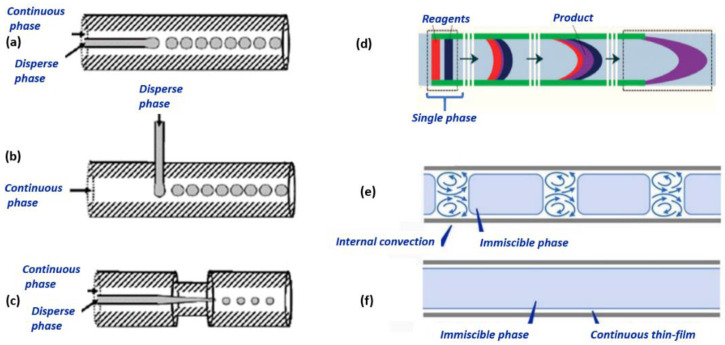
Common microfluidic flow types: (**a**) co-flow [96], (**b**) cross-flow [96], (**c**) flow-focusing [96], (**d**) continuous flow [97], (**e**) slug flow [98], and (**f**) annular flow [98]. Reprinted from open-access sources.

For a better understanding of microfluidic methods, the most common microreactor flow types are gathered in Figure 3.

### 3.3. Advantages over Classic Methods

As more and more synthesis reactions are moving towards microfluidic production, it is clear that there are several advantages in comparison to classic methods. In this context, Table 2 comprises these benefits in an organized manner.

### 3.4. Limitations and Challenges of Microfluidic Approaches

There is no doubt that microfluidic technology has many advantages compared to preexisting synthesis and testing methods. However, certain aspects become more pronounced when miniaturizing equipment down to the microscale, e.g., surface roughness, capillary forces, and chemical interactions between materials [101]. Hence, some specific challenges and limitations are to be considered.

The enhancement of material properties can cause unexpected experimental complications as the reactor behaves differently from traditional laboratory equipment [101]. The small dimensions impose a limitation on the nanoparticle production rate as the possible flow-rates do not compare with those from conventional bulk mixing methods [16,38]. In addition, the formation of undesired products due to side reactions is not completely solved by microreactors. However, secondary chemical reactions are minimized through the accurate control of reaction conditions, leading to much smaller amounts of by-products than in macroscale processes [108,109].

Moreover, the small diameters of the channels make them susceptible to clogging [11]. The solute concentration can be increased to solve the production rate issue, but this may lead to precipitation on channel walls, followed by particle growth inside the chip [38]. A similar effect is caused by the production of insoluble materials during polymerization reactions when very high molecular weights are obtained [8]. Nanoparticle agglomeration or formation of aggregates may also be behind channel blockage [14,38]. The effect is stronger at the wall surface due to the longer residence time induced by the laminar velocity profile [8]. Microchannel clogging remains a major concern in synthesis processes as it alters mixing and may result in experimental failure [11,14].

Another challenge consists of choosing the right device substrate, especially because many materials have poor solvent compatibility and low resistance to high temperature. In this respect, novel materials should be developed to manufacture reliable and cost-effective chips [11]. Furthermore, the manufacturing techniques, supply, and demand are not in favor of microfluidic industrialization, as there is a lack of large-scale production development [10,16,95]. To increase interest in mass production, purification and extraction processes should be improved and integrated with nanoparticle synthesis to create fully automated production [11].

## 4. Nanomaterials Synthesized through Microfluidic Methods

As nanotechnology is still in its infancy, nanoparticles’ production and application are expected to continuously improve [16]. In recent years, microfluidic methods were exploited to synthesize nanoparticles with different sizes, shapes, and surface compositions, with small size distribution, high drug encapsulation efficiency, prolonged circulation time, and heightened tumor accumulation [13,41]. Depending on the reaction conditions and finite products’ requirements, chips of various materials and geometries can be employed. Typical substrates include glass, silicon, metals, polymers, and ceramics, but the diversity and quality of materials are continuously increasing [10,14,104,110,111]. In terms of channel geometry, two main classes of devices can be distinguished: flow-focusing and T-junction [112].

To correlate these aspects with the synthesis methods and the obtained products, several research studies concerning nanomaterials synthesis through microfluidic methods were summarized in Table 3, Table 4, Table 5 and Table 6.

### 4.1. Inorganic Nanomaterials

Inorganic nanoparticles find use in various fields, ranging from electronics, energy, and textiles to biotechnology, bio-imaging, and bio-sensing. Most of these applications are based on materials such as gold, silver, silica, alumina, titanium oxide, and zinc oxide, but not exclusively [16,41,113].

Noble metal nanoparticles, such as gold, silver, and platinum, are of special interest in medical applications due to their size and shape properties [84,88]. Various metal nanoparticles of controlled size and structure can be synthesized in droplet-based microfluidic reactors via the reduction of metal ion precursors in the presence of stabilizing ligands [113].

Gold nanoparticles (Au NPs) were produced via microfluidic methods by several researchers, inspired by the outstanding properties and potential applications of this material. Generally, the reduction of a gold precursor takes place in the presence of different types of ligands and stabilizers. The use of strong reducing agents, such as sodium borohydride, ensures fast nucleation and small sizes of finite products. The reduction of gold ions fits in a timeframe of seconds, following fast kinetic crystallization at the nanoscale [16]. Spheres, spheroids, rods, and other various shapes can be obtained from spherical Au NP seeds (of less than 4 nm in size) by adjusting the concentrations of reagents, feed rates of individual aqueous streams, reduction potentials of the metal complex, and adsorbate binding strength [11,88].

Silver nanoparticles (Ag NPs) are another category of noble metal nanoparticles with properties much different from the bulk material [101]. Over the past decade, Ag NPs have been widely used, especially due to their antimicrobial, optical, and electrochemical properties [114]. The intrinsic features of AgNPs are in strong correlation with particle size, shape, composition, crystallinity, and structure, among which size and shape are the most important [103]. For this reason, the possibility for precise control within microfluidic devices increased the research interest in microreactor synthesis.

Zinc oxide nanoparticles (ZnO NPs) have drawn much attention recently in the field of nanomedicine, especially for tissue engineering, targeted drug delivery, contrast agents, and therapeutics against cancer [115]. To obtain high-quality ZnO NPs, their synthesis can be performed in microfluidic devices as well. The controlled production of ZnO NPs with well-defined physicochemical properties has already been demonstrated to be effective for various shapes, such as wires, spheres, rods, spindles, ellipsoids, and sheets [116].

Titanium oxide nanoparticles (TiO_2_ NPs) of uniform size can also be rapidly and economically produced in microreactors [117,118]. The synthesized particles have excellent photodegradation efficiency, rendering them suitable for environmental remediation applications [117].

Silica nanoparticles (SiO_2_ NPs) are also considered valuable in various fields, attracting interest in their microfluidic production [119]. One of the most important configurations for biomedicine purposes is mesoporous silica, a material of intensive research in recent years [120,121].

Magnetic nanoparticles (MNPs) are an important class of nanomaterials due to their unique properties, such as chemical stability, magnetic response, biocompatibility, and low cost [16]. These advantageous features created interest in the microfluidic production of MNPs to be further used for a wide range of applications in biomedicine-related fields, such as biomedical imaging (e.g., contrast-enhancing agents in magnetic resonance imaging), site-specific drug delivery, bio-sensing, diagnosis, biological sample labeling, and sorting [11,84,119,122,123]. Cobalt nanoparticles (Co NPs) are an example of MNPs with different properties depending on the crystal structure [119]. Other MNPs that have gained research attention are iron oxide nanoparticles (IONPs) [119]. These materials have promising properties required in nanomedical applications, making their microfluidic production a natural step towards enhancing IONPs quality [76].

Quantum dots (QDs) can be produced in microfluidic systems by miniaturizing the traditional synthesis methods, leading to high-quality, monodisperse particles [124]. Semiconductor QDs, in general, and Cadmium Selenite (CdSe) QDs, in particular, have attracted much interest in scientific research due to their tunable bandgap, narrow emission spectrum, high conductivity and mobility, and outstanding chemical and light stability [114]. Moreover, their tunable photoluminescence in the visible spectrum allows CdSe QDs to be used for biomedical purposes and optical-electronic applications [113].

Besides metallic-based nanoparticles, microfluidics has attracted recent interest for the synthesis of non-metallic materials, as well. One such example is represented by sulfur, the ability of which to inhibit bacteria and fungi makes these nanoparticles suitable for sterilization of food and utensils. The controllable particle size and uniform morphology attained through microfluidic technology improve sulfur nanoparticles’ bactericidal performance [125].

**Table 3:** Summary of inorganic nanomaterials synthesized via microfluidic approach.

Synthesis Product	Microreactor Type	Main Reagents/Materials	Synthesis Observations	Products Observations	Ref.
AuNPs	Passive PDMS-based chip	Chloroauric acid, borohydride (reducing agent), tri-sodium citrate (capping agent)	Room temperature; reaction time under 5 min	Average size of nanoparticles: 2 nm	[99]
AuNPs	PDMS-based chip with S-shaped channels	Gold seeds (prepared in advance by reducing HAuCl4 with NaBH4), silver nitrate, ascorbic acid	Sufficient mixing, precise flow rate control	Gold nano-bipyramids with controllable morphology	[126]
AgNPs	Continuous flow SPD made of SUS	Silver nitrate, L-ascorbic acid, soluble starch, poly(4-vinylpyridine)	Room temperature, intense mixing; a very thin fluid film forms on the rapidly rotating disc	Nanoparticles size is controlled through varying rotating speed	[127]
AgNPs	Droplet-based PDMS chip	Silver nitrate, tannic acid, trisodium citrate	Room temperature	Droplet size and residence time can be influenced by changes in flow rates and flow ratio between continuous and dispersed phases	[128]
AgNPs	Flow-focusing droplet-based PDMS chip	Silver nitrate, silver seeds (prepared in advance by a reaction of silver nitrate and sodium borohydride), pure water, trisodium citrate dihydrate, liquid paraffin	Temperature: 60 °C (to ensure seed growth within microdroplets)	Average size of the particles can be increased by increasing reaction time, temperature, and concentration of silver cations, and decreased by increasing seed concentration	[103]
ZnO NPs	SUS microreactor	Zinc sulfate and potassium hydroxyl solutions	Hydrothermal synthesis; temperature: ≈400 °C (maintained by an electric furnace); crystals were collected by filtrating the slurry solution and drying at 60 °C	Average diameter: 9 nm	[129]
ZnO nanostructures	Glass capillaries	Zinc acetate dihydrate, diethanolamine, zinc nitrate hexahydrate, methenamine, ammonium hydroxide solution	Dip-coating process for the seed layer deposition, combined with the continuous-flow chemical process	Different morphologies can be obtained on the inner wall of the capillary tubes	[130]
TiO_2_ NPs	Ceramic microchannel reactor with a glass cover	TTIP dissolved in 1-hexanol, distilled water, formamide	The reaction takes place at the stable interface between the two insoluble currents	Particles with a size of less than 10 nm; anatase polymorph	[118]
SiO_2_ nanofibers	Five-run spiral-shaped PDMS microreactor	CTAB, diluted ammonia, diluted TEOS	Room temperature	Mesoporous silica nanofibers; tunning of fibers aspect ratio by changing the flow rates or the concentrations of implied reagents	[120]
HSS with hierarchical sponge-like Pore sizes starting from several nanometers	Two-run spiral-shaped PDMS microreactor	CTAB, diluted ammonia, TMB, diluted TEOS	Rapid and efficient mixing	Well-defined spherical silica particles having an average diameter of ca. 1200 nm; hollow core and sponge-like large porous shell structure; pore size ranging from several nanometers to over 100 nm can be observed, depending on TMB concentration	[100]
Co NPs	Polymer-based chip	Cobalt chloride, tetrahydrofuran, lithium triethylborate (reducing agent), 3-(N,N-dimethyldodecylammonia)propanesulfonate (stabilizer)	Phase-controlled synthesis	Varying the experimental conditions such as flow rates, growth time and quenching procedure, the researchers managed to obtain mostly crystal structure	[131]
IONPs	Continuous flow spiral copper wire microreactor	Iron nitrate nonahydrate, sodium hydroxide, N-cetyl trimethyl ammonium bromide	Co-precipitation and reduction reactions; room temperature	The average particle size of IONPs decreased with an increase in the flow rate of the reactants, reaching an average particle size of 6 nm for a flow rate of 60 mL/h	[132]
CdSe QDs	PTFE micromixer chip	Cadmium oleate, Se-TOP solution	3–60 min incubation time; the faster growth rate in the microfluidic synthesis than in the bulk reaction	Higher absolute photoluminescence quantum yields than in bulk synthesis	[114]
SNPs	Two reactors: YMC and TMC	Sublimed sulfur, carbon disulfide (solvent), ethanol (anti-solvent)	Continuous anti-solvent precipitation process; a suspension is obtained at the outlet, requiring further spray drying to get SNP powders	Highly stable monodispersed sulfur nanoparticles with a size of 15–50 nm	[125]

### 4.2. Organic Nanomaterials

Microfluidic methods have also been employed for the synthesis of organic nanoparticles due to their potential use in pharmaceutical formulations [113]. This emerging technology is promising for improving treatment outcomes by enhancing the bioavailability and specificity of the therapeutic agent while reducing its toxicity [7,81].

Liposomes are of special interest, being efficient transport vehicles for in vivo applications, as hydrophilic drugs can be entrapped in their interior aqueous core while lipophilic and amphiphilic substances can be incorporated into the lipid bilayers [83,124]. Liposomes are highly efficient drug delivery systems due to their biocompatibility, enhanced drug encapsulation, and ease of surface modification [41]. Such systems achieve selective and sufficiently precise localization of the diseased site while also ensuring a slow and sustained release [124,133]. Such features are critically required for the treatment of chronic and acute disorders, including cancer, inflammatory disorders, or infectious diseases [134,135]. The challenge to produce liposome formulations with a defined or limitedly variable size [124] was overcome by microfluidic production, demonstrated since 2004 [41]. The most common approach is to synthesize liposomes in droplet-based microfluidic systems [81], but reproducible control of particle size and size distribution can be achieved in continuous-flow microfluidic devices as well [83].

Polymer-based nanoparticles (PNPs) synthesis within microfluidic devices is considered promising as well, as it offers improved control over size, size distribution, morphology, and composition of such particles [16,78]. Poly-(lactic-co-glycolic acid) nanoparticles (PLGA NPs), a polymer approved by the Food and Drug Administration (FDA), can be fabricated via a flow-focusing method in microchannels. Nanoparticles of this polymer can also be obtained using the droplet-based method by combining microfluidic droplet generation with solvent extraction techniques [41]. The synthesis of PLGA- poly-(ethylene glycol) nanoparticles (PLGA-PEG NPs) has been performed by nanoprecipitation in a hydrodynamic flow-focusing microchannel. The desired size, polydispersity, and drug loading can be achieved through the variation in flow rates, polymer composition, and polymer concentration [84]. A similar nanoprecipitation process was conducted to obtain polycaprolactone (PCL) nanoparticles, biodegradable entities with extensive potential for controlled drug delivery [136]. Some other polymers, such as heparin, chitosan, and hyaluronic acid, can be assembled in microfluidic devices for PNPs useful in the delivery and controlled release of drugs [41].

**Table 4:** Summary of organic nanomaterials synthesized via microfluidic approach.

Synthesis Product	Microreactor Type	Main Reagents/Materials	Synthesis Observations	Products Observations	Ref.
Liposomes	Microfluidic vertical flow-focusing device made of a thermoplastic material	Lipid, aqueous buffer	Continuous flow synthesis	Tunable size ranging from 80 to 200 nm; nearly monodispersed vesicles	[137]
Liposomes	SUS-derived V-shape mixer connected with Teflon tubing	1,2-distearoyl-sn-glycero-3-phosphocholine, cholesterol, N-(carbonyl-methoxypolyethyleneglycol 2000)-1,2-distearoyl-sn-glycero-3-phosphoethanolamine, ethanol, physiological saline	Tubing passed through a water bath at 25 °C	The size of liposomes is controlled by changing the relative flow rate of an ethanol solution of lipids and aqueous solutions	[138]
Liposomes	Ultrasound-enhanced microfluidic system	Egg phosphatidylcholine, cholesterol, PBS	The microfluidic chip was placed in the water-bath of a bath sonicator; Efficiently combined the advantages of microfluidic and sonication technologies	Flow rate ratio affects the particle size	[139]
PLGA NPs	Plus-shape flow-focusing microfluidic chip made of Teflon	PLGA dissolved in DMSO, PVA dissolved in distilled water	Nanoprecipitation (after injecting PLGA and PVA solutions to the microdevice, DMSO started to diffuse into the aqueous phase, and PLGA nanoparticles precipitated out)	Compared to batch synthesis, the obtained particles were more uniform and harmonious in size, more stable, monodisperse, and spherical	[140]
PEG-PLGA NPs	PI film microreactor with direct 3D flow-focusing geometry	PEG-PLGA polymers in acetonitrile, water	Performed at flash flow (11 ms of retention time in a unit microchannel)	Monodisperse PEG-PLGA nanoparticles with average diameters of 50 nm and 85 nm	[141]
PCL NPs	Glass microfluidic devices (with different confluence angles and channel dimensions)	Aqueous phase: PVA, Tween 80, Milli-Q water Organic phase: PCL, THF	Hydrodynamic flow-focusing method; controlled self-assembly process; non-solvent precipitation technique	Microchannels with shorter lengths produced smaller nanoparticles due to the shorter residence time of the particles in the mixing channel; a small confluence angle of 60° is more favorable for producing smaller nanoparticles	[136]
HA NPs	Glass cross-junction microchannel	Aqueous phase: sodium hyaluronate solution, ADH, EDCl, deionized water Organic phase: Ethanol, IPA, or acetone	pH of 6.0; crosslinked HA NPs were formed at the interface between the organic phase and water in a laminar flow inside a flow-focusing microchannel	The ability of the non-solvents to dehydrate hyaluronic acid decreases from ethanol, IPA, to acetone, while the mean diameter increases in the order of ethanol, IPA, to acetone	[142]

### 4.3. Active Pharmaceutical Ingredients

The pharmaceutical industry benefits from microfluidic approaches as they allow for cheaper, more effective, and more accessible production of drug formulations [143,144]. Enhanced control over reaction conditions and the excellent quality of the products are the main reasons behind several pharmaceutical companies’ decision to implement this technology as an alternative to hazardous exothermic power-intensive processes [145,146].

Up to date, various active pharmaceutical ingredients have been reportedly produced within microfluidic systems. Their list includes but is not limited to nitroglycerin [147], ibuprofen [146], lactose [148], aspirin [148], telmisartan [149], hydrocortisone [150], indomethacin [151], danazol [152], cefuroxime axetil [153], piroxicam [154], piracetam [154], and carbamazepine [154].

**Table 5:** Summary of active pharmaceutical ingredients synthesized via microfluidic approach.

Synthesis Product	Microreactor Type	Main Reagents/Materials	Synthesis Observations	Products Observations	Ref.
Nitroglycerin	Acrylic chip	Glycerol, nitric acid, sulfuric acid (catalyst)	The reaction rate is controlled by the diffusion process and the medium viscosity; the higher the concentration of the reactants, the higher the probability of particle collisions	The use of the microchannel produces more nitroglycerin reaction products compared to using the batch reactor system	[147]
TEL NPs	Silicone tube mounted over a glass plate	Aqueous phase: various polymers (PVP K-30, PVP K-90, HPMC, Poloxamer 407, and Poloxamer 188) dispersed in water Organic phase: telmisartan dissolved in acetone and dichloromethane	Continuous microfluidic nanoprecipitation process; rapid nucleation; diffusion-controlled mixing	The particle size for the five investigated polymers increased in the order d_Poloxamer407_ < d_PVPK−30_ < d_HPMC_ < d_PVPK−90_ < d_Poloxamer188_; recrystallized TEL nanoparticles showed clear and nearly uniform shape surface morphology	[149]
Hc NPs	YMC	Hc, HPMC, sodium lauryl sulfate;	Room temperature	Hc dispersions in the range of 80–450 nm; mean particle size can be changed by adjusting the experimental parameters and design of microreactors	[150]
Indomethacin nanocystals	Droplet-based PDMS chip	Indomethacin, amaranth, agarose, paraffin liquid, anhydrous ethanol, propidium iodide	Stable hydrogel droplets with uniform size were continuously generated on a microfluidic chip; the concentrations of the drug, the ratios of solvent and antisolvent in each stable hydrogel droplet could be well-controlled by regulating the flow rates of syringe pumps	Crystals of indomethacin with different morphologies were formed in the hydrogel droplets on the chip	[151]
Danazol NPs	YMC	Danazol, ethanol (solvent), deionized water (antisolvent)	Nanoprecipitation; antisolvent temperature: 4 °C	Mean size of 364 nm	[152]
CFA NPs	YMC	CFA, acetone (solvent), isopropyl ether (antisolvent), SDS, deionized water	Rapid mixing, immediate precipitation; the formed suspension is filtrated, and the precipitate is dried at 40 °C under vacuum	Nanoparticles with narrow PSD, size-dependent, and enhanced dissolution rate	[153]
Piroxicam	72-well microfluidic platform made of thin layers of PDMS and X-ray transparent COC	Piroxicam dissolved in acetonitrile:methanol mixture (1:1 volume ratio)	Drug-seeds were generated off-chip, then harvested, placed in a tissue homogenizer glass tube, and mixed with API solution. The seed-solution was introduced on-chip and left for incubation	The seeds confirmed as form I yielded well-formed rectangular prisms	[154]
Piracetam	72-well microfluidic platform made of thin layers of PDMS and X-ray transparent COC	Piracetam dissolved in methanol	Drug-seeds were generated off-chip, then harvested, placed in a tissue homogenizer glass tube, and mixed with API solution. The seed-solution was introduced on-chip and left for incubation	The 1:5 and 1:10 micro-seed dilution experiments yielded largely but poorly formed and twinned crystals	[154]
Carbamazepine	72-well microfluidic platform made of thin layers of PDMS and X-ray transparent COC	Carbamazepine dissolved in acetonitrile	Drug-seeds were generated off-chip, then harvested, placed in a tissue homogenizer glass tube, and mixed with API solution. The seed-solution was introduced on-chip and left for incubation	The seeding method directed the crystallization towards the predominant formation of form III crystals	[154]

### 4.4. Hybrid and Composite Nanomaterials

Multifunctional entities can be formed by loading inorganic nanomaterials in polymer particles [16,155], benefiting from their components’ synergic properties. Microfluidic devices also offer the possibility to produce complex hybrid nanostructures in simple processes, shorter times, and controlled reaction conditions, which would otherwise be unattainable [16]. Thus, composites comprising two inorganic materials can be efficiently synthesized in microfluidic reactors [155] to match the requirements of applications in the biomedical field, especially as fluorescent biological labels [156].

Another promising combination is the creation of lipid–polymer nanoparticles for drug delivery [11]. What makes these hybrid nanomaterials so appealing is the possibility of encapsulating drug molecules in both the polymeric core and the lipid shell through microfluidic methods [41,78]. Additionally, drug-loaded particles can also be obtained through microfluidic techniques, resulting in products of reduced size and higher drug-loading capacity [157].

Other interesting delivery systems that can be synthesized in microfluidic devices are lipid nanoparticles loaded with nucleic acids. In the context of the COVID-19 pandemic, the fabrication of monodispersed lipid vesicles became essential for the encapsulation of messenger RNA (mRNA) required in vaccines’ formulation [158,159,160]. Particularly, this is achieved through the mixing of an ethanol phase (containing the hydrophobic lipids) and an aqueous phase (containing mRNA in a buffer, e.g., acetic acid, at pH 4) in a droplet-based microreactor [159,161].

Moreover, artificial leukocytes and lipoproteins can be fabricated via assembling proteins with lipid molecules. Thus, by assembling phospholipids with apolipoproteins within a microfluidic device, high-density lipoproteins were mimicked [41].

**Table 6:** Summary of hybrid and composite nanomaterials synthesized via microfluidic approach.

Synthesis Product	Microreactor Type	Main Reagents/Materials	Synthesis Observations	Products Observations	Ref.
ZnS-coated CdSe	Multi-step continuous microfluidic system	TOP-Se stock solution (prepared from Se powder and TOP), Cd(CH_3_COO)_2_, stearic acid, TOPO, diethylzinc, bis(trimethylsilyl) sulfide	CdSe solution preparation: Cd(CH3COO)_2_ was added to stearic acid and heated at 130 °C. (TOPO) was then added under a nitrogen flow. After the solution was cooled to below 100 °C, it was mixed with the TOP-Se stock solution ZnS solution preparation: diethylzinc and bis(trimethylsilyl) sulfide were dissolved in TOP, then mixed with melted TOPO; CdSe preparation: Oil bath at 300 °C Coating step: Oil bath at 220 °C	Control the particle size and layer thickness by simply adjusting the residence time	[162]
PtSn intermetallic nanocrystals	Microfluidic reactor with segmented regions (heating plate and water bath)	Pt(acac)_2_, PEG400, SnCl_4_⋅5H_2_O, EG	A PMMA bottle with pressures by pressure regulated N_2_ was used as the collection vial; products were collected by centrifugation process, washed with ethanol and water three times, and dried overnight at 60 °C	Pure PtSn intermetallic phase is demonstrated in products formed in reactions at more than 250 °C	[163]
Polystyrene-encapsulated IONPs	Continuous flow microfluidic device	For the polymer nano-emulsion: styrene (monomer), SDS (surfactant), hexadecane (Ostwald ripening inhibitor), potassium peroxydisulfate (initiator) For the magnetite nanoparticles: anhydrous ferric chloride, ferrous chloride tetrahydrate, ammonium hydroxide, octane, oleic acid	Microfluidic elongational flow method; magnetite particles obtained by co-precipitation were further coated with oleic acid and dried to obtain a powder; polymer nano-emulsion is left overnight in an oven at 70 °C becoming a stable colloidal suspension, by thermal polymerization	Excellent product quality, homogenous composite particle size distribution; encapsulation of a lower content of iron oxide nanoparticles but with a smaller size than those encapsulated by batch processes	[164]
Ag NP-loaded chitosan particles	PMMA chip with a cross-junction channel	Chitosan, silver nitrate, glucose, sodium hydroxide	A one-step mechanism involving the reduction of Ag NPs and solidifying the chitosan particles in emulsions simultaneously	The size of products can be controlled to achieve a narrow size distribution; various uniform chitosan microparticles impregnated with Ag NPs were successfully obtained	[165]
Liposomal-AuNP hybrids	Automated microfluidic system	AuNPs, toluene, chloroform, methanol, HSPC, DSPE-PEG_2000_, DPH, PBS	The methanolic mixture containing both the lipids and the AuNPs was mixed with an aqueous solution (PBS, pH 7.4); once prepared, the hybrids were dialyzed for 24 h to remove traces of methanol and then were concentrated in a viva-spin column	Homogeneous size distribution, smaller polydispersity index, and three times higher loading capacity than when using the traditional methodology	[134]
Liposome-hydrogel hybrid NPs	Microchannels in a silicon substrate anodically bonded to a glass borosilicate cover	1,2-dipalmitoyl-sn-glycero-3-phosphocholine, cholesterol, dihexadecyl phosphate, isopropanol, 1,1′-dioctadecyl-3,3,3′,3′-tetramethy-lindodicarbocyanine perchlorate, poly(N-isopropylacrylamide), PBS	Microfluidic mixing controlled by hydrodynamic focusing	Narrowly dispersed populations of lipid-hydrogel hybrid nanoparticles; size range appropriate for targeted delivery and controlled release applications	[166]
PEG-cHANPs	Microfluidic chip with an X-junction configuration	HA-SH, PEG-VS, pure acetone (non-solvent)	Hydrodynamic Flow Focusing; one-step process (nanoprecipitation); temperature: 4 °C	Average size: 140 nm; Accurate control over final nanoparticle properties by simple tuning of focused stream width and process parameter adjustment	[167]
PEGylated PLCL	Two microfluidic chips: a cross-flow chip with an X-shaped mixing junction (2D laminar flow-focusing) and a micromixer featuring a YMC	3,6-dimethyl-1,4-dioxane-2,5-dione (lactide), CL, stannous 2-ethylhexanote (catalyst), different initiators (1-dodecanol, a MeO-PEG-OH, and a 4-armed star PEG-OH)	Ring-opening polymerization at 140 °C; continuous flow nanoprecipitation	Nanoparticle formulations were produced with Z-average sizes in the range of 30–160 nm; smaller particles were obtained with a YMC (30–120 nm), especially for the PEGylated polyesters (30–50 nm), whereas the cross-flow chip systematically produced larger particles (80–160 nm)	[168]
PLGA NPs coated with a muco-penetrating stabilizer (Pluronic F68)	Cross-channel microreactor	Aqueous phase: Pluronic F68, water Organic phase: PLGA, acrylonitrile	Nanoprecipitation; NPs suspension was left overnight for organic solvent evaporation, followed by two centrifuge washes and redispersion with Milli-Q water to remove excess stabilizer	Particles had a tunable hydrodynamic diameter ranging from 40 nm to 160 nm	[169]
HA-functionalized lanthanide-doped KGdF_4_ NPs	Two PMMA chips (one for each synthesis step)	GdCl_3_·6H_2_O, EuCl_3_·6H_2_O, Ce(NO_3_)_3_·6H_2_O, TbCl_3_·6H_2_O, KF·2H_2_O, DEG, sodium hyaluronate	Two steps: (1) synthesis of Ln3^+^-doped KGdF4 nanoparticles (room temperature, ultrafast, continuous process) and (2) functionalization with HA (via electrostatic adsorption)	The synthesized nanoparticles show good uniformity, high biocompatibility, targeted cellular uptake, photoluminescence, and magnetic resonance properties	[170]
PLGA NPs loaded with EFV	Borosilicate glass capillaries on a glass slide	Aqueous phase (outer fluid): PLGA, dimethyl sulfoxide, EFV Organic phase (inner fluid): Tween^®^ 80 solution	Nanoprecipitation; after production, particles were washed three times with ultrapure water and recovered by ultrafiltration	Reduced NP size, comparable polydispersity, less negative zeta-potential, higher EFV association efficiency, and higher drug-loading than in the conventional approach	[157]
CoQ_10_-MITO-Porter	Microfluidic device incorporating a baffle mixer (named iLiNP device)	Aqueous phase: PBS Organic phase: lipids (DOPE, SM, DMG-PEG 2000, and STR-R8), CoQ_10_, and ethanol	Lipids in ethanol and PBS were mixed to form a suspension, which was further dialyzed for at least 2 h	Homogeneously distributed, small-sized CoQ_10_-MITO-Porter that efficiently internalized into cells and accumulated in mitochondria	[144]
Amphiphilic HFR bioconjugates	Solvent-resistant microfluidic device made of low molecular weight perfluoropolyether	UFH dissolved in formamide, N-(3-dimethylaminopropyl)-N’-ethylcarbodiimide hydrochloride dissolved in formamide, aminated RA dissolved in DMF	Ultrafast reaction time; single-step synthesis	Bioconjugates with high drug coupling ratio; nanoparticles likely have a core-shell structure composed of a hydrophobic inner core containing aggregated RA molecules and a hydrophilic UFH or HF shell; average size: 130–141 nm	[171]
HMCS with encapsulated PTX	TMC PDMS microfluidic device	HMCS, PTX mixed with an acidic solution, basic water	Physiological pH (7.4)	HMCS nanoparticles with high concentrations of PTX	[172]
Ribavirin-loaded PLGA NPs	Continuous flow microfluidic reactor system	Aqueous phase: ultrapure water containing ribavirin Organic phase: PLGA dissolved in acetonitrile, acetone, or DMSO	No precipitate was noticed in the micro-channels during the flow-focusing experiments; NPs were recovered by centrifugation, washed several times with non-solvent solution, centrifuged, and freeze-dried	Drug-loaded NPs smaller than 100 nm	[173]
Ketoprofen-encapsulated PMMA NPs	Three chips: TMC, HPIMM, and K-M micromixer	Ketoprofen; mannitol; cremophor ELP; methanol; THF; SDS; methyl methacrylate; copper (I) bromide; 1,1,4,7,10,10-hexamethyltriethylenetetramine; 2-ethyl bromoisobutyrate; ultrapure water	Micromixer-assisted nanoprecipitation; nanoprecipitation started immediately inside the mixing chamber when both fluids (polymer solution including ketoprofen and ultrapure water) were brought into contact	Size range: 100–210 nm; the size of the nanoparticles decreases with the water flow rate; the TMC produces the largest nanoparticles while the K-M micromixer generates the smallest ones	[174]

## 5. Conclusions

Nanomaterials can have many different shapes and chemical compositions, which means that their properties (such as size, design, solubility, surface modifications, charge, deformability, etc.) can be tailored to meet specific application requirements. However, conventional synthesis methods do not offer precise control over reaction parameters, affecting the desired outcomes. By precise manipulation of nanoliter volumes, microfluidic devices enable the synthesis of high-quality nanoparticles, drug carrier systems, active pharmaceutical ingredients, composite nanomaterials, and even cells. As there is a long list of advantages of microfluidic production over conventional synthesis, it is expected that this technology would exponentially gain interest in developing new materials, processes, and functionalities. Moreover, translating microfluidics to large-scale production should be considered to make this technology more popular and industrially appealing. Hence, research should also be directed towards standardization, automation, and high-throughput.

## Figures and Tables

**Table 1:** Conventional methods for the synthesis of nanoparticles and nanocomposites.

Synthesis Products	Synthesis Method		Description	Refs.
Nanoparticles	Co-precipitation		Simultaneous occurrence of nucleation, growth, coarsening, and/or agglomeration processes	[17,43]
	Hydrothermal synthesis		Chemical reactions between substances found in a sealed, heated solution above the ambient temperature and pressure	[17,43]
	Inert gas condensation		Metals undergo evaporation in an ultrahigh vacuum chamber filled with He or Ar at high pressure, collide with the gas, and condense into small particles, forming nanocrystals in the end	[17,44]
	Sputtering		Ejection of atoms from the surface of a material by bombardment with energetic particles	[17,45]
	Microemulsion		An isotropic, macroscopically homogeneous, and thermodynamically stable solution containing a polar phase, a nonpolar phase, and a surfactant; reactant exchange occurs during the collision of droplets within the microemulsion	[17,46,47,48]
	Microwave-assisted		Synchronized perpendicular oscillations of electric and magnetic fields produce dielectric heating throughout the material at the molecular/atomic level	[48,49]
	Laser ablation		Removing material from a (usually) solid surface by irradiating it with a laser beam	[17,48,50]
	Sol-gel		5-step method: hydrolysis of precursors, polycondensation (gel formation), aging (continuous changes in the structure and properties of the gel), drying, and thermal decomposition	[51]
	Ultrasound		Ultrasonic cavitation induced by irradiating liquids with ultrasonic radiation	[17,52]
	Spark discharge		An abrupt electric discharge occurs when a sufficiently high electric field creates an ionized, electrically conductive channel through a normally insulating medium, thus producing a highly reactive soot	[17,53]
	Template synthesis		Uniform void spaces of porous materials are used as hosts to confine the synthesized nanoparticles as guests	[17,54]
	Biological synthesis		Synthesis using natural sources, avoiding any toxic chemicals and hazardous byproducts, usually with lower energy consumption	[55]
Nanocomposites	Spray pyrolysis		A thin film is deposited by spraying a solution on a heated surface, upon which the constituents react to form a chemical compound	[17,56]
	Infiltration		A preformed dispersed phase is soaked in a molten matrix metal, which fills the space between the dispersed phase inclusions	[17,57]
	Rapid solidification		Rapid extraction of thermal energy to include both super heat and latent heat during the transition from a liquid state at high temperature to a solid material at room temperature	[17,58]
	High energy ball milling		High mechanical forces provide energy for the activation and occurrence of a chemical reaction	[59]
	Vapor deposition (VD)	Chemical VD	The substrate is exposed to volatile precursors that react and/or decompose on its surface to produce the desired deposit	[17,48,60,61]
		Physical VD	The material goes from a condensed phase to a vapor phase and then back to a thin film condensed phase	[17,62]
	Colloidal method		Under controlled temperature and pressure, different ions are mixed in a solution to form insoluble precipitates	[47,63]
	Powder process		Compression, rolling, and extrusion are used to obtain a compact mass that is further sent to a sintering furnace	[17,64]
	Polymer precursor		A polymeric precursor is mixed with the matrix material, undergoes pyrolysis in a microwave oven, thus generating the reinforcing particles	[17,65]
	Melt blending		Melting of polymer pellets to form a viscous liquid followed by the use of high shear force to disperse the nanofillers	[60,66]
	Solution mixing		Dispersion of nanofiller in a polymer solution by energetic agitation, controlled evaporation of the solvent, and composite film casting	[17,67]
	In situ intercalative polymerization		Polymer formation occurs between the intercalated sheets of clay	[17,68,69]
	In situ formation and sol-gel		A multi-step process including the embedding of organic molecules and monomers on sol-gel matrices followed by the introduction of organic groups by the formation of chemical bonds, resulting in situ formation of a sol-gel matrix within the polymer and/or simultaneous generation of inorganic/organic networks	[17,70]

**Table 2:** Advantages of microfluidic systems synthesis.

Advantages	Observations	References
high reproducibility	- reduced batch-to-batch variation - reproducible composition, structure, and physicochemical properties	[7,11]
narrow size distribution	- the polydispersity index can go as low as 0.02	[7,11,38]
tunable particle size	- reported sizes from 2 nm to 1200 nm	[11,38,99,100]
improved controlled features of nanoparticles	- improved control over nanoparticle crystal structure - synthesis of smaller mean particle size	[7,8,84]
well-controlled heat transfer	- owing to the large surface-to-volume ratio - possibility of fast heating and cooling of reaction mixtures - temperature homogeneity - requirement of only a small heat capacity	[7,11,16,84,101]
well-controlled mass transfer	- the small dimensions (micrometer scale) enable homogeneous mixing - in devices with laminar flows, concentration gradients are precisely controlled by varying channel length or relative flow velocities of the input fluid streams	[11,16,84,96,102]
efficient tunable mixing	- efficient mixing achievable in less than 60 ms	[7,16,38,84,103]
reduced reagent consumption	- pico-to-nano liter reagent amounts	[7,38,101]
short reaction time	- in the order of minutes	[84,101,104]
controllable residence time	- by controlling the length and geometry of the microchannels	[11,16]
rapid change of experimental conditions	- within microseconds	[84]
cost-effective	- less raw materials and energy input are required, reducing synthesis costs - possibility of automation decreases the need of manpower and labor associated costs	[7,101,104,105]
high throughput	- higher percent yields compared to conventional reactors, as the precise control over reaction parameters allows better selectivity towards the desired synthesis products	[7,14,104]
reduced generation of chemical wastes	- less by-product formation due to uniform processing conditions	[101,104,106]
compact systems	- more functionality in less space - combining several steps (preparation, analysis, synthesis, functionalization, purification) in a single chip	[84,101]
new reaction pathways	- reactions can be carried out more aggressively (e.g., performing highly exothermic reactions or using extreme temperatures can be done without the need of cryogenic systems required at macroscale) - microfluidic devices can be used when a proposed reaction situation is otherwise unattainable (e.g., selective fluorination and perfluorination of organic compounds, on-site and on-demand synthesis of positron emission tomography tracers)	[16,104,107]
safer operational environment	- spill is negligible in case of reactor failure - minimized explosions and leakages of harmful compounds - ease of containing	[14,16,104]

## Data Availability

Not applicable.

## Abbreviations

ADH	adipic hydrazid
AgNPs	silver nanoparticles
API	active pharmaceutical ingredient
AuNPs	gold nanoparticles
CdSe QDs	cadmium selenite quantum dots
CFA	cefuroxime axetil
CL	ε-caprolactone
COC	cyclic olefin copolymer
CoNPs	cobalt nanoparticles
CoQ10	coenzyme Q10
CoQ10-MITO-Porter	coenzyme Q10 encapsulated in a MITO-Porter liposome
CTAB	cetyltrimethylammonium
DEG	diethylene glycol
DMF	dimethylformamide
DMG-PEG 2000	1,2-dimyristoyl-rac-glycero-3-methoxypolyethylene glycol-2000
DMSO	dimethylsulfoxide
DOPE	1,2-dioleoyl-sn-glycero-3-phosphoetanolamine
DPH	1,6-diphenyl-1,3,5-hexatriene
DSPE-PEG2000	1,2-distearoyl-sn-glycero-3-phosphoethanolamine-N-[amino(polyethylene glycol)-2000]
EDCl	chloride carbodiimide
EFV	efavirenz
EG	ethylene glycol
HA NPs	hyaluronic acid nanoparticles
HA-SH	thiolated hyaluronic acid
Hc	hydrocortisone
HF	unfractionated heparin–folic acid conjugate
HFR	heparin–folic acid–retinoic acid
HMCS	hydrophobically modified chitosan
HPIMM	High-pressure interdigital multi-lamination micromixer
HPMC	hydroxypropylmethylcellulose
HSPC	hydrogenated soy phosphatidylcholine
HSS	hollow spherical silica
iLiNP	invasive lipid nanoparticle production
IONPs	iron oxide nanoparticles
IPA	isopropyl alcohol
MeO-PEG-OH	alpha-methoxy-omega-hydroxy poly(ethylene glycol)
PBS	phosphate buffered saline
PCL NPs	polycaprolactone nanoparticles
PDMS	polydimethylsiloxane
PEG-cHANPs	pegylated crosslinked hyaluronic acid nanoparticles
PEG-OH	hydroxy poly(ethylene glycol)
PEG-PLGA NPs	polyethylene glycol–poly(lactic-co-glycolic acid) nanoparticles
PEG-VS	polyethylene glycol–vinyl sulfone
PI	Polyimide
PLCL	poly (d,l-lactic acid-*co*-caprolactone)
PLGA NPs	poly(lactic-co-glycolic acid) nanoparticles
PMMA	polymethylmethacrylate
Pt(acac)2	platinum (II) bis(acetylacetonate)
PTFE	polytetrafluoroethylene
PTX	paclitaxel;
PVA	polyvinyl alcohol
PVP	polyvinylpyrrolidone
RA	retinoic acid
SDS	sodium dodecyl sulfate
SM	sphingomyelin;
SNP	sulfur nanoparticles
SPD	spinning disc processor
STR-R8	stearylated R8
SUS	stainless steel
TEL	telmisartan
TEOS	tetraethyl orthosilicate
THF	tetrahydrofuran
TiO2 NPs	titanium dioxide nanoparticles
TMB	1,3,5-trimethylbenzene
TMC	T-type microchannel
TOP	trioctylphosphine
TOPO	trioctyl phosphine oxide
TTIP	titanium tetraisoproxide
UFH	unfractionated heparin
YMC	Y-type microchannel
ZnO NPs	zinc oxide nanoparticles
